# A new HInT in the publishing panorama: an interesting opportunity to those working on health innovation and technologies

**DOI:** 10.3205/hta000131

**Published:** 2019-09-24

**Authors:** Antonio Migliore, Hans-Peter Dauben, Iñaki Gutierrez Ibarluzea, Brendon Kearney, Lisa Kurylowicz, Roberta Joppi

**Affiliations:** 1 Ufficio HTA: Innovazione e Sviluppo a supporto delle Regioni, Agenzia nazionale per i servizi sanitari regionali (Agenas), Rome, Italy; 2 Institute of Health Economics & Medical Outcome Research, University of Applied Sciences RFH, Cologne, Germany; 3 Basque Foundation for Health Innovation and Research Torre BEC, Barakaldo, Bizkaia Basque Country, Spain; 4 Department of Haematology, Royal Adelaide Hospital, Adelaide, Australia; 5 Clinical Research & Drug Assessment Unit Local Health Authority of Verona – Veneto Region, Verona, Italy

## Editorial

Dear readers,

GMS Health Innovations and Technologies (GMS HInT) is an open access electronic journal offered by EuroScan international network e.V. (EuroScan) and published by German Medical Sciences (GMS).

Born in 1997 as a working group of public agencies from several countries and formally established as a network in 1998, EuroScan acquired the status of a legal entity based in Germany in 2017, becoming a non-profit scientific association that operates at an international level on the basis of not for profit institutions from the whole world. EuroScan activities aim to collect and share information on health technologies in their life cycle to support decision-making on their adoption, appropriate use and need of re-assessment. EuroScan is also the main global forum for sharing and developing methods for the early identification and pre-assessment of emerging, new and obsolete health technologies. 

Within its activities, it was felt by the board of EuroScan that there was still a gap to be filled to reach its goals. As a scientific association, there was a need to share methods and experiences with others out of the boundaries of the association. In this sense, the board thought that a dedicated journal would be the ideal vehicle to reach these goals. When the opportunity arose to take responsibility for the journal GMS Health Technology Assessment (GMS HTA) that was edited by DIMDI (German Institute of Medical Documentation and Information) since 2005, we were delighted. To reflect the new aims and direction of Euroscan and the journal, we decided to change the journal’s name to GMS Health Innovation and Technologies (GMS HInT). There are some continuities with GMS HTA. For example, the particular interest in health technologies and evidence-based healthcare, in addition to some members of the GMS HTA editorial board continuing their commitment and pusuit on the GMS HInT editorial board. 

GMS HInT looks at health technologies from the lifecycle approach, focusing on emerging and new technologies as well as obsolete ones. It aims to bring experiences and issues from real policy or clinical/health environments on the use of innovative health technologies to a broad audience of international healthcare professionals and the science community. Thus, GMS HInT contributes to the knowledge pool of evidence-based health care.

This journal is not competing with other journals in this field of knowledge; it tries to be complementary by presenting descriptions of the successful or ineffective management of innovations and health technologies throughout their lifecycle in different parts of the world. These lessons have been part of the continuous information sharing among EuroScan members during its years of existence, but have not yet been shared with a broader audience in a systematic way. GMS HInT also gives an opportunity to academia, government officials and health professionals in general to share their local experiences and lessons learnt for the benefit of others. Its electronic open access format will guarantee continuous publication and dissemination of articles without having the limits of the periodical issuing, meanwhile allowing peers to share their methods and experiences internationally.

In addition to original articles in English, it is possible to include appendices in other languages. These appendices are not part of the scientific publication and are therefore not subject to quality assurance procedures. Moreover, full-text articles in German can be considered for publication if an abstract in English is provided by the authors.

GMS HInT is an international peer-reviewed journal that fully operates in accordance to the international rules on biomedical publishing and publication ethics. Following the registration of the name change – from GMS HTA to GMS HInT, GMS HInT will be indexed in PubMed and full articles will be archived in the PubMed Central repository. The journal will also be listed in the following repositories and supporting editorial services:

- DIMDI
- DOAJ
- JournalSeek
- LIVIVO
- Open J-Gate
- Socolar

GMS HInT applies the double-blind unbiased peer review, including an internal review by section editors and external reviewers, who are experts on the topic.

The typical target audience of GMS Health Innovations and Technologies is composed of (but not limited to) academia, healthcare professionals, healthcare service researchers, innovators and developers and early awareness and alert units.

The publication priorities of GMS Health Innovations and Technologies are methods and outputs in the field of

- horizon scanning and disinvestment
- local real-world experiences of management of emerging, new, and obsolete health technologies
- health models
- studies on big data

We are excited about beginning this editorial adventure and we are looking forward to your contributions.

Antonio Migliore, Hans-Peter Dauben, Inaki Gutierrez Ibarluzea, Brendon Kearney, Lisa Kurylowicz & Roberta Joppi

